# Vitrification and Nanowarming. Is this the Future of Kidney Transplantation

**DOI:** 10.3389/ti.2023.11948

**Published:** 2023-11-08

**Authors:** Sarah A. Hosgood, Michael L. Nicholson

**Affiliations:** Department of Surgery, University of Cambridge, Cambridge, United Kingdom

**Keywords:** kidney, cryopreservation, nanowarming, preservation, normothermic machine perfusion, vitrification

Transplanting an organ within a set timeframe to facilitate allocation and matching is a challenge. The commonest method of kidney preservation is to simply flush the organ with cold preservation solution at the time of retrieval to remove the blood and cool it to below 4°C. The kidney is then stored in ice until transplantation. This technique of static cold storage reduces the metabolic demand and requirement for oxygen to limit the rate of deterioration. The storage time is kept below 24 h to reduce cold ischaemic injury and risk of graft loss [1].

The recent paper by Han et al. [2] and colleagues at the University of Minnesota addresses the issue of time by successfully demonstrating an innovative technique of vitrification and nanoparticle rewarming which allowed rodent kidneys to be preserved for up to 100 days prior to successful transplantation [2]. This is a significant step forward towards the concept of organ banking. The ability to bank organs would allow a more elective approach to transplantation, with better matching and allocation and tailored induction protocols for the recipients.

Cryopreservation is the storage of cells or organs at very low temperatures. It was first attempted in the 1800s but with limited success. One of the major problems is the formation of ice crystals within the cell due to instant freezing and thawing [3, 4]. Ice crystals disrupt the cellular membranes causing deformities in the cell structure [3, 4]. Increases in solute concentration also occur as ice crystals form intracellularly during cooling [3, 4]. To reduce the formation of ice crystals two protective actions are needed [5–7]: slow increments in the speed of freezing and rewarming and the use of cryopreservation agents such as dimethyl sulfoxide (DMSO), glycerol or polyethylene glycol [5–7]. Cryopreservation agents increase the porosity of the cellular membrane and interact strongly with water through hydrogen bonding to reduce the freezing point and the formation of ice crystals [8]. The formation of solid water with an irregular, amorphous structure is known as vitrification. This is achieved with the use of a cryopreservation solution and the appropriate cooling rate. During vitrification cells or organs are cooled from 37°C to −150°C in a stable, ice-free, glass-like state [8]. To reduce toxicity, vitrification mixtures are added in a stepwise manner. This allows the successful storage of cells in a solid phase at supercool temperatures to halt biochemical processes without the formation of ice [8].

Han et al. demonstrated that rodent kidneys can be vitrified and rewarmed to sustain kidney function for 30 days after transplantation [2]. This is a significant step forward from the work of Fahy et al. in 1984 who reported a single rabbit kidney transplant after vitrification for 8 min [9]. They found that vitrification and rewarming were hampered by inadequate tissue cooling due to reduced quantities of cryopreservation solution to avoid toxicity and by the formation of ice crystals upon rewarming [9].

Han et al. overcame these issues with the administration of iron oxide nanoparticles throughout the organ vasculature with a newly formulated cryopreservation solution called VMP [2]. The iron oxide nanoparticles are silica and polyethylene glycol (PEG) coated to increase the stability of the cryopreservation solution and provide biocompatibility and organ washout. The organ was vitrified by first perfusing or loading the kidney gradually with VMP solution. Iron oxide nanoparticles were perfused at the final step of loading. The kidney was then placed in a controlled rate freezer and cooled at a rate faster than the cryopreservation solution’s critical cooling rate to enter a stable glassy state. The vitrified organ was then stored at −150°C. Rewarming occurred by placing the kidney in a radio frequency coil that induced alternating magnetic fields from an electric current flowing through the coil. The magnetic fields generated an oscillatory response in the nanoparticles that generated heat throughout the system. The radio frequencies penetrate tissues without causing damage. Histologically the kidneys had no evidence of ice formation. Several kidneys were tested during a period of *ex vivo* normothermic machine perfusion with an oxygenated acellular solution. Vascular resistance was comparative to fresh control kidneys, and they demonstrated metabolic function by consuming oxygen and glucose.

In the final series of experiments the nanowarmed rodent kidneys were transplanted and the animals recovered for 30 days post-transplant. There was some initial graft dysfunction but after day 14 post-transplant renal function recovered to that of healthy controls. At day 30, renal function was similar to fresh control kidneys. Histology showed some focal tubular necrosis and hyaline change but intact basement membranes and vasculature. This study is a significant breakthrough in the application of cryopreservation using biochemical and engineering principles to overcome the toxicity associated with cryopreservation solutions and rewarming in a unified manner using nanoparticle technology to prevent crystallisation. Nonetheless, the question remains as to whether this technology can be applied in clinical kidney transplantation.

The rising incidence of chronic renal failure has increased the burden on kidney transplantation. The use of donation after circulatory death (DCD) and expanded criteria donors has increased rates of transplantation but the gap between supply and demand is growing. There is also a high rate of kidney discard due to insufficient organ quality [10]. Although cryopreservation extends the time that kidneys can be preserved several questions remain, the most crucial being can this be applied to human kidneys? The perfusion of cryopreservation solution into the rat kidney has solved the problem of damaging ice crystal formation during freezing but it is questionable whether this can be scaled up to a human kidney. Can 200 g of tissue have its water replaced by cryopreservation solution quickly enough to protect the tissue deep in the kidney, especially the medulla where blood flow is low? Furthermore, the use of iron oxide nanoparticles to allow magnetic rewarming is a novel and clever idea but again, can this be scaled up to a human kidney? Would the central deep tissues of the kidney be warmed efficiently and is there any potential toxicity of iron oxide? Moreover, if the technique was successfully applied to kidneys from marginal donors, would they be of sufficient quality for transplantation? With cryopreservation techniques a post-thaw reduction in viability is inevitable.

Organ perfusion technologies are a fast-developing area of research [11]. Recent focus has been on developing normothermic or subnormothermic machine perfusion (NMP) techniques [12, 13]. These preserve a level of cellular metabolism and restore function to avoid or limit cold ischaemic injury. In the liver, NMP has been used to extend the preservation interval to days rather than hours [14]. Experimental evidence with human organs suggests that prolonged perfusion may also be possible in the kidney [15]. Prolonged NMP may extend the preservation interval to allow better matching and allocation and also provide an opportunity to treat the kidney to repair damaged cells. The administration of regenerative or gene therapies is gaining interest in this area [16, 17]. One other advantage of NMP is the ability to assess the quality of the kidney to determine suitability for transplantation. Although, the exact assessment criteria have not yet been defined, basic functional perfusion parameters such as flow, appearance and urine production can provide a measure of kidney quality [18, 19].

The concept of organ banking using cryopreservation and nanoparticle rewarming would certainly ease constraints on allocation and allow better matching. However, it is likely that upon rewarming an assessment of viability would be needed. Rather than a competing technology, NMP could be complementary and used in conjunction with cryopreservation to assess the quality before transplantation.

Vitrification and nanoparticle rewarming is an exciting new approach that offers many advantages in transplantation and the work by Han et al. provides proof of principle that it can be achieved. The next step of this research would be to study it in human kidneys.
